# Normobaric Hypoxic Cardiac Rehabilitation: Comparative Effects of Training at 2000 m and 3000 m Simulated Altitude in Post-Myocardial Infarction Patients

**DOI:** 10.3390/jfmk10040444

**Published:** 2025-11-18

**Authors:** Agata Nowak-Lis, Tomasz Gabryś, Zbigniew Nowak, Anna Konarska-Rawluk, Dominika Grzybowska-Ganszczyk, Radosław Chruściński

**Affiliations:** 1 Department of Physiotherapy, Jerzy Kukuczka Academy of Physical Education, ul. Mikołowska 72a, 40-065 Katowice, Poland; z.nowak@awf.katowice.pl (Z.N.); anko@int.pl (A.K.-R.); dominikagrzybowska@yahoo.com (D.G.-G.); 2 Sport Centrum Faculty of Education, University of West Bohemia, 301 00 Pilsen, Czech Republic; tomaszek1960@o2.pl; 3 Faculty of Health Science, University of Applied Science, 48-300 Nysa, Poland; radek.chruscinski@gmail.com

**Keywords:** normobaric hypoxia, cardiac rehabilitation, myocardial infarction, training

## Abstract

**Background:** Coronary artery disease remains the leading cause of morbidity and mortality in developed countries. Despite advances in treatment and standard rehabilitation, conventional programs may be monotonous and insufficiently engaging. Normobaric hypoxia, simulating high-altitude conditions, has emerged as a potential method to enhance cardiovascular adaptations in post-myocardial infarction (MI) patients. **Objective:** This study aimed to compare the efficacy and safety of exercise-based cardiac rehabilitation performed under normobaric hypoxia corresponding to altitudes of 2000 m and 3000 m above sea level in patients after MI treated with percutaneous coronary intervention (PCI). **Methods:** A total of 61 male post-MI patients (mean age 60.4 ± 8.9 years) were randomized into two groups: training under simulated altitudes of 2000 m (*n* = 35) or 3000 m (*n* = 26). The 22-day program consisted of interval ergometer sessions. Pre- and post-intervention assessments included cardiopulmonary exercise testing (CPET), echocardiography, and tissue Doppler imaging (TDI). **Results:** Both groups demonstrated significant improvements in exercise tolerance. Training at 2000 m significantly increased test duration (r = 0.735) and peak heart rate (r = 0.467). At 3000 m, additional benefits were observed, including improvements in metabolic equivalent (r = 0.861), peak oxygen consumption (d = 0.81), and reduction in respiratory exchange ratio (r = 0.682). Intergroup analysis revealed moderate differences favoring the 3000 m group in MET, breathing frequency, and RER. Echocardiography showed beneficial remodeling in both groups, with improvements in LV dimensions, ejection fraction, and MAPSE. Notably, training at 2000 m resulted in more consistent echocardiographic benefits compared to 3000 m. **Conclusions:** Cardiac rehabilitation under normobaric hypoxia is effective and safe in stable post-MI patients. Training at 3000 m provides greater improvements in exercise tolerance, while 2000 m confers more favorable effects on cardiac structure and function. These findings suggest that moderate hypoxic exposure (2000 m) may represent an optimal balance between efficacy and safety in post-MI rehabilitation.

## 1. Introduction

Coronary artery disease remains one of the leading causes of morbidity and mortality in developed countries. Advances in the treatment of acute coronary syndromes, particularly primary percutaneous coronary intervention with stent implantation, have significantly improved survival rates among patients following myocardial infarction. Consequently, an increasing number of patients are discharged from the hospital in good clinical condition and are capable of engaging in various forms of physical activity.

However, standard cardiac rehabilitation programs applied in the second phase of recovery are often conservative. Although their effectiveness has been repeatedly confirmed and documented [1,2,3,4], they are frequently monotonous and not tailored to the actual capabilities of patients, resulting in decreased motivation and discontinuation of regular exercise. Therefore, there is a growing interest in more functional and engaging methods that reflect daily activities and provide conditions conducive to long-term cardiovascular adaptation. One potential approach is endurance training conducted under normobaric hypoxia, simulating a high-altitude environment. This issue has increasing practical significance due to the popularity of mountain tourism and winter sports among this patient population. Existing research indicates that short-term exposure to altitudes of up to 3000–3500 m above sea level may be relatively safe in individuals with stable coronary artery disease, provided that exercise tolerance has been adequately assessed by a stress test [5,6,7,8]. Attention is also drawn to the necessity of several days of acclimatization prior to undertaking more intense physical activity.

The ESC and PTK guidelines provide detailed recommendations regarding physical activity in patients after acute coronary syndromes; however, they do not address hypoxia or exposure to high-altitude environments [9]. Meanwhile, available observational and experimental studies suggest that hypoxic conditions may promote beneficial cardiovascular adaptations, including vasodilation, improved endothelial function, and reduced atherosclerosis progression. According to Burtscher et al. [10], adaptation to hypoxia occurs mainly through the activation of transcription factors HIF-1α and HIF-2α, which regulate the expression of genes responsible for angiogenesis (VEGF), erythropoiesis (EPO), and metabolic adjustments that enhance oxygen utilization. Hypoxia also induces mitochondrial changes and activates antioxidant pathways (including the Nrf2-Keap1 system), which help to reduce oxidative stress and support cellular adaptation to low-oxygen conditions. Moderate or intermittent hypoxia may exert a hormetic effect by stimulating protective and neuroprotective mechanisms. However, prolonged or excessive hypoxia can trigger inflammatory processes and cellular damage. Therefore, the overall effect depends on the dose, duration, and frequency of hypoxic exposure. Clinical studies in this area are limited and typically involve small patient cohorts, largely due to the high organizational costs and the need for medical support in high-altitude settings or laboratories equipped with hypoxic chambers [11,12]. Despite these limitations, the results obtained are promising and indicate the potential of this approach as a complement to conventional cardiac rehabilitation.

## 2. Aim of the Study

The aim of this study was to compare the efficacy and safety of an exercise program conducted under normobaric hypoxia corresponding to altitudes of 2000 m and 3000 m above sea level in patients after myocardial infarction who underwent percutaneous coronary intervention, and to determine which altitude confers greater clinical benefit. The following research questions were formulated:

1. Which environmental conditions (2000 m vs. 3000 m above sea level) more favorably influence changes in exercise tolerance assessed by cardiopulmonary exercise testing (CPET)?
2. Does training under normobaric hypoxic conditions (2000 m and 3000 m above sea level) affect changes in cardiac hemodynamic parameters?

## 3. Materials and Methods

### 3.1. Study Population

The study included 61 male patients following myocardial infarction who underwent percutaneous coronary intervention with stent implantation. Participants (volunteers) were referred from the Cardiology Department of the University Hospital in Katowice between October 2023 and March 2024. Initially, 65 patients (61 men and 4 women) participated in the study. Due to the small number of female participants, only male patients were included in the analysis. The characteristics of the study population are presented in Table 1.

The mean age of the patients, type of myocardial infarction, and number of implanted stents were similar in both groups. All participants took part in the second phase of cardiac rehabilitation. Due to the high risk of adverse effects associated with hypoxic conditions, only patients after an uncomplicated myocardial infarction who achieved ≥7 METs during the initial treadmill exercise test were included. The test was terminated either upon reaching the target submaximal heart rate (85% of HRmax) or due to fatigue.

Inclusion criteria:

- History of uncomplicated myocardial infarction, at least 4 weeks post-event,
- Male and female patients aged 35–75 years,
- Eligibility for cardiac rehabilitation according to Model A (≥7 METs),
- Informed consent to participate,
- No active inflammatory diseases or uncontrolled non-cardiac comorbidities.

Exclusion criteria:

- Unstable coronary artery disease,
- Recent myocardial infarction (within 4 weeks),
- Chronic heart failure,
- Arrhythmias or conduction abnormalities on ECG,
- Treatment-resistant hypertension,
- Positive stress test,
- Peripheral artery disease of the lower limbs,
- Thrombosis/embolism,
- Chronic obstructive pulmonary disease (COPD),
- Anemia,
- Musculoskeletal disorders preventing exercise testing,
- SARS-CoV-2 infection,
- Lack of consent to participate.

**Figure 1:**
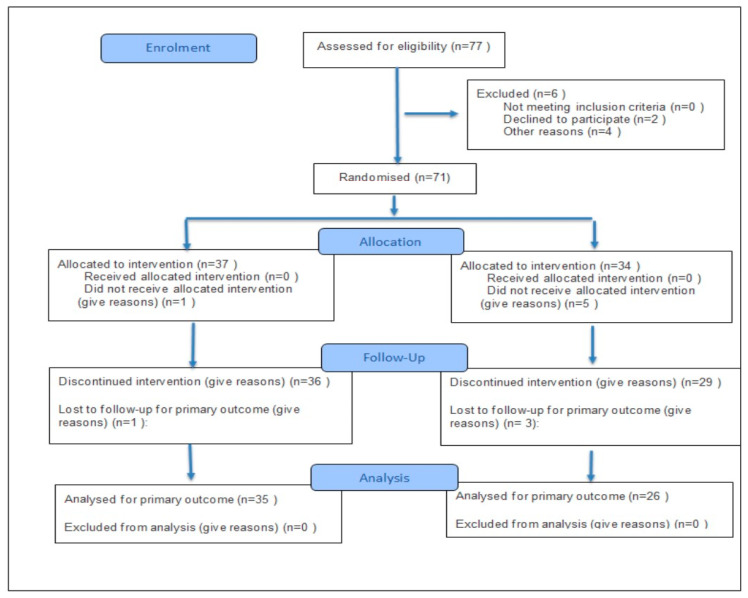
Flow diagram of the study.

Assignment to the training groups under normobaric hypoxia corresponding to 2000 m and 3000 m above sea level was random, using the eCRF.biz computerized clinical trial generator (https://www.ecrf.biz.pl, accessed on 13 November 2025). Participants could withdraw from the study at any time. According to the study protocol, patients’ pharmacological treatment was optimized and consistent with current guidelines for coronary artery disease and post-myocardial infarction management. Medication doses remained unchanged during the experiment. Informed consent for participation was obtained from all subjects involved in the study. Figure 1 presents a flow diagram of the study.

### 3.2. Study Methods

Normoxic assessments were conducted twice for each patient: before the start of the outpatient rehabilitation program (Phase II) and after completion of the 22-day training cycle (days 23–24 of the experiment). The procedure included: day 1—cardiopulmonary exercise test (CPET) on a treadmill; day 2—echocardiographic examination of the heart. All procedures were performed according to ESC [13] and PCS [14] guidelines.

Exercise tolerance was assessed using a submaximal spiroergometric test (85% of HRmax calculated as 220–age) on a treadmill, employing the standard six-stage Bruce protocol: Stage 1: 2.7 km/h, 10% incline; Stage 2: 4.0 km/h, 12%; Stage 3: 5.5 km/h, 14%; Stage 4: 6.8 km/h, 16%; Stage 5: 8.0 km/h, 18%; Stage 6: 8.8 km/h, 20%.

The following parameters were analyzed: Test duration [min], Distance [m], Energy cost (MET), Resting (HR_rest_) and peak heart rate (HR_peak_) [bpm], Resting and peak blood pressure (SBP, DBP) [mmHg], Peak ventilation (VE) [L/min], Breathing frequency (BF) [L/min], Peak oxygen consumption (VO_2peak_/kg) [mL/min/kg], Respiratory exchange ratio (RER).

During the test, each patient was monitored using a 12-lead ECG. The test was concluded once all measured parameters returned to baseline values. Spiroergometric parameters were assessed using the CORTEX METAMAX 3B gas analyzer with a Pulsar treadmill (H/P Cosmos, Nussdorf-Traunstein, Germany). The system was turned on at least 20 min prior to testing and calibrated according to the manufacturer’s recommendations, including a 3 L gas syringe (5530 lot, Hans Rudolph, Inc., Shawnee, MO, USA) volume calibration. Tests were terminated upon reaching the target heart rate (85% HRmax) or due to fatigue, with no other (non-physiological) reasons for termination reported.

For patient safety, a certified medical rescuer with a full resuscitation kit and portable AED was present throughout the tests. All patients took their prescribed medications on the test day.

Echocardiographic evaluation was performed by a cardiologist trained in echocardiography according to ESC guidelines [15], using a GE Vivid Q (General Electric, Wuxi, China) device. Left ventricular ejection fraction (LVEF) was determined using the Simpson 2D method with a sector transducer M5Sc-D and GE v.204 software.

Measured parameters included: LVEDd—left ventricular end-diastolic diameter (mm), LVESd—left ventricular end-systolic diameter (mm), LVESV—left ventricular end-systolic volume (mL), LVEDV—left ventricular end-diastolic volume (mL), LVEF—left ventricular ejection fraction (%).

Tissue Doppler imaging (TDI) indices were also assessed: A wave—atrial contraction velocity [m/s], E wave—early ventricular filling velocity [m/s], e’ lateral—early diastolic velocity of lateral mitral annulus [m/s], e’ septal—early diastolic velocity of septal mitral annulus [m/s], E/e’—ratio of mitral inflow velocity to mitral annular early diastolic velocity, E/A—ratio of early to atrial mitral inflow velocities, TAPSE—tricuspid annular plane systolic excursion, MAPSE—mitral annular plane systolic excursion.

### 3.3. Training Protocol

Endurance training under normoxic and hypoxic conditions was conducted according to ESC recommendations, daily from Monday to Friday in the morning. During the experiment, in addition to patients and two researchers, a medical rescuer was present in the hypoxic chamber with full resuscitation equipment. Training was performed on Kettler ergometers (Kettler Ergo C10, Ense-Parsit, Germany).

Prior to entering the chamber, blood pressure, heart rate, and oxygen saturation were measured under normoxic conditions. Patients then underwent a 30 min acclimatization period inside the chamber (breathing and circulatory exercises in a seated position). Post-acclimatization measurements were repeated, and the training session commenced. Exercise intensity was individualized based on the initial treadmill test. Interval training with progressive load increases every three days was applied: Warm-up—5 min (initial 20 W, increased by 5 W every three days), main session—35 min (intervals: 4 min exercise/3 min low-load recovery; initial 20 W, increased by 5 W every three days), cool-down—5 min (20 W), rest—10 min (stretching, breathing exercises). Training intensity was set at 60–80% of the maximal workload achieved during the initial CPET test, corresponding to 70–85% of HRmax. The workload was increased every three days, taking into account the subjective rating of perceived exertion (Borg scale 12–14).

Training chamber conditions:

- 2000 m a.s.l.: O_2_ 16.8%, temperature 19.9 °C, CO_2_ 1514 ppm, humidity 33.2%, atmospheric pressure 993 hPa
- 3000 m a.s.l.: O_2_ 14.8%, temperature 21 °C, CO_2_ 1586 ppm, humidity 33.6%, atmospheric pressure 985 hPa

Due to water loss during chamber exercise, each patient had access to bottled still water, and during the experiment, patients did not participate in any other physiotherapeutic interventions.

The study was approved by the Bioethics Committee (No. 7/2017, 18 May 2017) and registered in the Australian New Zealand Clinical Trials Registry (ACTRN12619001350112, 1 October 2019).

### 3.4. Statistical Analysis

Data analysis was conducted using OpenOffice 4.0.1 (Apache Software Foundation, Delaware, DE, USA), StatSoft Statistica (StatSoft Poland, Kraków, Poland), and GraphPad Prism 6.07 (GraphPad Software, San Diego, CA, USA). Normality was assessed with the Shapiro–Wilk test and frequency histograms. Variance homogeneity was evaluated using the Brown–Forsythe test.

**Intragroup comparisons:** Parametric: repeated measures ANOVA for normally distributed variables with homogeneous variances, Student’s *t*-test, effect size (Cohen’s d: <0.2 small; 0.2–0.5 moderate; 0.5–0.8 large; >0.8 very large). Non-parametric: Wilcoxon test, effect size r (0.1 small; 0.3 moderate; 0.5 large). **Intergroup comparisons:** Parametric: Student’s *t*-test, Cohen’s d, non-parametric: Mann–Whitney U test, effect size r (0.1 small; 0.3 moderate; 0.5 large).

## 4. Results

*Spiroergometric Exercise Test*

Table 2 presents a comparative analysis of the exercise test results obtained before (I) and after (II) the 22-day training program conducted under normobaric hypoxia corresponding to altitudes of 2000 and 3000 m a.s.l.

In all patient groups, exercise tolerance improved after completion of the rehabilitation program. Assessment of the extent of changes with respect to the environmental conditions under which training was conducted showed that in the normobaric hypoxia condition corresponding to 2000 m a.s.l., statistically significant improvements were observed in test duration (r = 0.735, large effect) and peak heart rate (HR_peak_) (r = 0.467, moderate effect). Among patients training under normobaric hypoxia corresponding to 3000 m a.s.l., significant changes were observed in test duration (d = 1.12, very large effect), metabolic equivalent (MET) (r = 0.861, large effect), peak oxygen consumption (VO_2peak_) (d = 0.81, large effect), respiratory exchange ratio (RER) (r = 0.682, large effect), and peak heart rate (HR_peak_) (r = 0.482, moderate effect).

The intergroup comparison (Table 3) revealed statistically significant differences in metabolic equivalent (MET) (r = 0.301, moderate effect), breathing frequency (BF) (d = 0.272, moderate effect), and respiratory exchange ratio (RER) (r = 0.290, moderate effect).

Statistical analysis demonstrated favorable changes in the assessed hemodynamic parameters and tissue Doppler imaging (TDI) measurements in all participants, regardless of the environmental conditions in which the rehabilitation program was conducted (Table 4).

In the group training under normobaric hypoxia corresponding to 2000 m a.s.l., statistically significant changes were observed in: left ventricular end-diastolic diameter (LVEDd) (d = 0.55, moderate effect), left ventricular end-systolic diameter (LVESd) (d = 0.43, moderate effect), left ventricular ejection fraction (LVEF) (r = 0.42, moderate effect), early diastolic velocity of the septal mitral annulus (e’ septal) (r = 0.627, large effect), mitral annular plane systolic excursion (MAPSE) (r = 0.428, moderate effect).

In the group training under normobaric hypoxia corresponding to 3000 m a.s.l., significant changes were observed in: LVEDd (d = 0.61, moderate effect), early diastolic ventricular filling velocity (E wave) (r = 0.514, large effect), e’ septal (d = 0.52, large effect), MAPSE (r = 0.421, moderate effect).

The intergroup analysis revealed one statistically significant difference regarding the mitral annular plane systolic excursion (MAPSE) (r = 0.33, moderate effect) (Table 5).

## 5. Discussion

### 5.1. Cardiopulmonary Exercise Testing (CPET)

One of the most important diagnostic procedures used in cardiac rehabilitation is the assessment of the body’s response to gradually increasing physical workload. In addition to providing electrocardiographic data indicating the presence or absence of myocardial ischemia during exercise, this method also allows for the evaluation of a patient’s exercise capacity and the intensity of effort at which ischemia occurs, often accompanied by characteristic chest pain. In most patients, the test is symptom-limited, meaning it is terminated in the presence of fatigue, dyspnea, dizziness, angina pectoris, pain in other regions (e.g., lower limbs), or when the ECG recording shows clear evidence of ischemia and/or arrhythmias. It is generally recommended that exercise testing should always precede the initiation of the next stage of cardiac rehabilitation [16]. Moreover, it is advised that a follow-up exercise test be performed after completion of a cardiac rehabilitation program to assess its outcomes.

In our study, CPET was used to evaluate exercise tolerance in patients after myocardial infarction, before and after 22 endurance training sessions conducted under environmental conditions simulating altitudes of 2000 and 3000 m a.s.l. (normobaric hypoxia). The effects of training were assessed, and comparative analysis identified which condition was more effective in improving exercise tolerance and cardiorespiratory fitness. The effectiveness of training programs conducted in normoxia for post-MI patients has been repeatedly confirmed [17,18,19,20]. However, only few studies have described the influence of environmental conditions associated with normobaric hypoxia on the course and outcomes of post-infarction rehabilitation.

One of the key factors influencing the development of collateral circulation is hypoxia, which, in combination with physical exercise, induces angiogenesis and arteriogenesis, thereby promoting the formation of new vessels [21,22]. It has been demonstrated that the presence of well-developed collateral circulation in the infarct-related artery territory improves left ventricular function, reduces anginal symptoms, enhances exercise tolerance, improves quality of life, long-term prognosis, and decreases infarct size [23].

Analysis of exercise test results in patients who underwent their rehabilitation program under hypoxic conditions, regardless of altitude (2000 or 3000 m a.s.l.), revealed an appropriate physiological response to the existing conditions. Despite significant oxygen reduction in the training environment, final exercise tests showed substantial improvements in exercise tolerance. Comparative analysis indicated that training at 3000 m a.s.l. was more effective, with significant improvements in test duration (*p* = 0.000), metabolic equivalent (MET) (*p* = 0.000), peak oxygen uptake (VO_2peak_) (*p* = 0.000), reduced respiratory exchange ratio (RER) (*p* = 0.001), and increased peak heart rate (HR_peak_) (*p* = 0.020). Training conducted at 2000 m a.s.l. was also effective, with significant improvements in test duration (*p* = 0.000) and HR_peak_ (*p* = 0.024).

The beneficial effects of hypoxia combined with exercise were also demonstrated by Glazachev et al. [24], who applied intermittent hypoxia (10% O_2_) with 3 min hyperoxic recovery intervals in post-MI patients. In their study, 46 patients were divided into two groups: one exposed to hypoxia only (without exercise), and the other undergoing a standard rehabilitation program in normoxia. Both groups showed significant improvements in exercise tolerance and left ventricular hemodynamic parameters, demonstrating that hypoxic exposure alone can enhance cardiorespiratory performance.

According to some authors, training at high altitudes (>2000–3500 m a.s.l.) may lead to a blunted or even decreased maximal heart rate response [25,26], although Mourot et al. [27] reported, based on clinical trials, that the HRmax decline may be less pronounced under normobaric compared to hypobaric hypoxia. In our study, a slight but statistically significant increase in HR_peak_ was observed.

Training in a hypoxic chamber forces the body to adapt to reduced oxygen availability similar to conditions at high altitude. Consequently, the cardiopulmonary system is challenged to work harder while oxygen supply is limited, enhancing oxygen transport capacity and muscle metabolism. Hypoxia also contributes to increased maximal oxygen uptake (VO_2_max) during both submaximal and maximal exercise, thereby raising aerobic capacity. In our study, an increase in VO_2peak_ was noted only in patients training at 3000 m a.s.l., although it did not reach statistical significance. A similar effect was observed by Burtscher et al. [28], who demonstrated that intermittent hypoxia improves exercise tolerance by enhancing stress resistance and oxygen delivery. A three-week period of passive, short-term intermittent hypoxia exposure (14–10% O_2_) resulted in increased aerobic capacity and exercise tolerance in 16 men (8 with and 8 without prior myocardial infarction). After hypoxic exposure, peak oxygen uptake increased compared to normoxic conditions (*p* < 0.001). This improvement was closely related to elevated arterial oxygen content following hypoxia. Resting heart rate decreased, which was also observed in our study. Moreover, in our patients, as previously mentioned, a significant reduction in peak RER was found in the 3000 m group (*p* = 0.001).

An RER ≥ 1.10 is generally considered a marker of maximal effort during CPET, but exceeding this threshold is not in itself an indication to terminate the test. An RER < 1.00 at peak exercise reflects submaximal effort and may also be observed in patients with pulmonary limitations [29]. However, not all patients with heart failure are able to achieve optimal RER values during symptom-limited CPET, due to skeletal muscle abnormalities (structural, morphological, or metabolic), respiratory muscle fatigue leading to ventilatory impairment, adverse drug effects, or excessive fatigue [30]. In our study, RER values at peak exercise were within the expected physiological range both before and after training in both groups. In the 2000 m group, the baseline RER was 1.1 (submaximal effort), and after training, it remained at a similar level. In the 3000 m group, however, RER decreased significantly (*p* = 0.001), suggesting greater effectiveness of training under these conditions for both the cardiovascular and respiratory systems.

In summary, all patients demonstrated improvements in exercise tolerance. However, the extent of these changes depended on the environmental conditions of training (normoxia vs. hypoxia). Reduced oxygen availability in the training environment had a decisive influence on the achieved results. The lowest oxygen level (14.5%) was associated with the greatest increase in aerobic capacity.

### 5.2. Echocardiographic Assessment

Echocardiography, alongside cardiopulmonary exercise testing, is a fundamental procedure preceding qualification for cardiac rehabilitation and planning of exercise intensity. It allows for non-invasive evaluation of key parameters that determine the risk associated with physical training. However, the influence of exercise training on myocardial function and structure in post-myocardial infarction (MI) patients remains not fully elucidated. Most studies suggest that exercise training in patients with heart failure does not substantially improve left ventricular (LV) function. Nonetheless, some authors argue that an increase in cardiac pump performance in response to training cannot be excluded [31,32].

The relationship between exercise capacity and LV functional indices is weak. Preserved exercise capacity does not necessarily rule out impaired systolic LV function. Patients with reduced ejection fraction (EF) at rest are often able to perform moderate-intensity exercise. Exercise tolerance depends not only on LV function but also on age, training status, comorbidities, and even psychological condition [33]. Improvements in exercise performance with training are mediated primarily by peripheral adaptations, including enhanced perfusion, skeletal muscle metabolism and ultrastructure, and an increased anaerobic threshold. Based on literature data, it cannot be unequivocally stated that exercise training improves LV systolic function [34].

Following MI or coronary artery disease, myocardial damage and structural remodeling occur. Increased LV end-diastolic dimension (LVEDd), LV end-systolic dimension (LVESd), altered LV volumes, and neurohormonal dysregulation (overactivation of the renin–angiotensin–aldosterone system and catecholamines) contribute to impaired LV diastolic function. Consequently, a decline in LVEF is often observed, serving as a key marker of global LV contractility and an important prognostic parameter in post-MI patients [35]. LVEF is also widely used to monitor the effectiveness of comprehensive cardiac rehabilitation programs.

In our study, echocardiographic assessment of LV indices relevant to rehabilitation efficacy demonstrated overall beneficial changes in both groups, regardless of training conditions (hypoxia simulating 2000 or 3000 m a.s.l.). It is important to note that most changes occurred within the normal reference ranges for each parameter. A significant reduction in LVEDd was observed in both groups (2000 m: *p* = 0.002; 3000 m: *p* = 0.004). In contrast, Nowak et al. [11] did not observe such changes after a 4-week cardiac training program in post-MI patients, possibly due to sample size differences. LVESd decreased significantly only after training at 2000 m a.s.l., a finding consistent with the earlier work of Nowak et al. [13]. Mean LVEF increased significantly only in the 2000 m group, although baseline values were within the normal range (≥50%) in all participants. Previous studies have demonstrated that patients with reduced baseline EF (<50%) tend to normalize this parameter more rapidly during training, likely due to enhanced angiogenesis and arteriogenesis, ultimately improving LV systolic function [35,36]. In patients with preserved EF, exercise training typically induces only modest changes.

Tissue Doppler imaging (TDI) analysis in our study revealed no significant changes in most measured indices. Similarly, in a cohort of 48 men after MI who underwent a 4.5-month training program (16 interval sessions on a cycle ergometer followed by 24 gymnastic sessions), no significant improvements in TDI parameters were found. Conversely, Yu et al.) demonstrated that 8 weeks of training in 127 post-MI patients with moderate LV diastolic dysfunction resulted in significant improvements in mitral inflow velocities (E, A, and E/A ratio) [37]. Skaluba and Litwin), analyzing 121 patients with suspected CAD and preserved EF, reported that an E/E’ ratio > 10 was a strong independent predictor of reduced exercise tolerance [38]. In our study, the mean E/E’ ratio remained within the normal range (<10) before and after training in all groups (normoxia, 2000 m, and 3000 m).

While no significant TDI changes were noted under normoxic training, hypoxic training at both 2000 m and 3000 m resulted in significant improvements in e’ septal velocity (*p* = 0.0002 and *p* = 0.013, respectively) and MAPSE (*p* = 0.039 and *p* = 0.043, respectively). These findings highlight the potential effectiveness of hypoxia-based training. However, direct comparisons with other studies are limited, as most available research has been conducted in healthy individuals or athletes rather than post-MI patients.

Training in moderate normobaric hypoxia at an altitude equivalent to 2000 m a.s.l. (FiO_2_ ≈ 16.8%) more effectively improved left ventricular function parameters, whereas training at 3000 m a.s.l. (FiO_2_ ≈ 14.8%) led to a greater increase in exercise tolerance. Both programs were safe and well tolerated, which supports their potential use in phase II of cardiac rehabilitation. However, at 3000 m a.s.l., the limited oxygen availability may have reduced exercise intensity and cardiac workload, while moderate hypoxia at 2000 m a.s.l. provided an optimal environment for cardiopulmonary adaptation without excessive physiological stress [28,39].

Training under simulated hypoxic conditions may serve as a controlled supportive component in post-infarction rehabilitation programs, especially for patients with limited ability to exercise outdoors.

### 5.3. Study Limitations

A major limitation of the study was the lack of a control group performing the same training under normoxic conditions. However, the aim of the study was not to compare the effects of training in different environmental conditions (hypoxia vs. normoxia), but to compare two different parameters of normobaric hypoxia (2000 vs. 3000 m above sea level). The study served as a pilot phase preceding a planned randomized controlled trial that will include a normoxic group. Only men participated in the study. During the initial qualification stage, four women applied (Figure 1—Declined because of “Other reason”). However, due to the small number of female participants, the study was standardized, and only men were included. Future research is planned to include women, as well as to re-examine the participants 6 and 12 months after the completion of the experiment (follow-up).

## Figures and Tables

**Table 1:** Characteristics of the studied groups (assessment of homogeneity).

	2000 m a.s.l. (*n* = 35)	3000 m a.s.l. (*n* = 26)
Age years	60.48 ± 8.54	60.30 ± 9.48
STEMI	16 (45.7%)	12 (46.15%)
NSTEMI	19 (54.3%)	14 (53.85%)
Number of stents		
1 stent	18 (51.4%)	14 (53.8%)
2 stents	11 (31.4%)	9 (34.6%)
≥3 stents	6 (17.2%)	3 (11.6%)

STEMI—*ST-Elevation Myocardial Infarction*, NSTEMI—*Non-ST-Elevation Myocardial Infarction*.

**Table 2:** Spiroergometric test results.

Parameters		2000 m a.s l.	*p*		3000 m a.s.l.	*p*
**time [min]**	**I**	9.920 ± 1.889	**0.000** **r = 0.735**	**I**	9.733 ± 1.944	**0.000** **d = 1.12**
	**II**	11.225 ± 2.297		**II**	11.273 ± 2.344	
**MET [mL/kg/min]**	**I**	7.734 ± 1.197	0.214	**I**	7.857 ± 0.993	**0.000** **r = 0.861**
	**II**	7.954 ± 1.443		**II**	8.565 ± 1.086	
**VE** **[L/min]**	**I**	86.611 ± 18.820	0.654	**I**	85.615 ± 18.789	0.574
	**II**	87.782 ± 18.874		**II**	88.726 ± 18.230	
**VO_2peak [mL/min/kg]_**	**I**	27.060 ± 4.219	0.360	**I**	27.533 ± 3.504	**0.000** **d = 0.81**
	**II**	27.622 ± 5.128		**II**	29.069 ± 3.769	
**BF [L/min]**	**I**	34.388 ± 6.277	0.412	**I**	34.569 ± 4.382	0.387
	**II**	35.428 ± 5.221		**II**	35.269 ± 4.834	
**RER**	**I**	1.161 ± 0.092	0.824	**I**	1.069 ± 0.103	**0.001** **r = 0.682**
	**II**	1.165 ± 0.081		**II**	1.008 ± 0.101	
**HR_rest_** **[L/min]**	**I**	71.714 ± 9.122	0.094	**I**	69.961 ± 7.738	0.527
	**II**	70.000 ± 8.815		**II**	68.769 ± 6.707	
**HR_peak_ [L/min]**	**I**	134.028 ± 14.015	**0.024** **r = 0.467**	**I**	136.769 ± 13.706	**0.020** **r = 0.482**
	**II**	138.571 ± 17.293		**II**	139.346 ± 10.673	

**MET**—metabolic equivalent, **VE**—peak minute ventilation [L/min], **VO_2peak_/kg**—peak oxygen consumption per kilogram body weight [mL/min/kg], **BF**—breathing frequency [breaths/min], **RER**—respiratory exchange ratio, **HR_rest_**—resting heart rate [bpm], **HR_peak_**—peak heart rate [bpm], **d**—Cohen’s d (effect size), **r**—effect size for the Wilcoxon test.

**Table 3:** Intergroup analysis.

	2000 m a.s.l. vs. 3000 m a.s.l.
**time [min]**	*p* = 0.585
**MET [mL/kg/min]**	***p* = 0.018, r = 0.301**
**VE [L/min]**	*p* = 0.947
**VO_2peak [mL/min/kg_**	*p* = 0.133
**BF [L/min]**	***p* = 0.033. r = 0.272**
**RER**	***p* = 0.024 r = 0.29**
**HR_rest_ [L/min]**	*p* = 0.619
**HR_peak_ [L/min]**	*p* = 0.502

**MET**—metabolic equivalent, **VE**—peak minute ventilation [L/min], **VO_2peak_/kg**—peak oxygen consumption per kilogram body weight [mL/min/kg], **BF**—breathing frequency [breaths/min], **RER**—respiratory exchange ratio, **HR_rest_**—resting heart rate [bpm], **HR_peak_**—peak heart rate [bpm], **r**—effect size for the Wilcoxon test.

**Table 4:** Comparison of echocardiographic and tissue Doppler imaging (TDI) parameters before and after training, depending on the altitude at which the program was conducted.

Parameters		2000 m a.s.l.	*p*		3000 m a.s.l.	*p*
**LVEDd** **[mm]**	**I**	49.285 ± 4.968	**0.002** **d = 0.55**	**I**	50.730 ± 4.911	**0.004** **d = 0.61**
	**II**	45.516 ± 7.546		**II**	48.538 ± 4.658	
**LVESd** **[mm]**	**I**	34.314 ± 6.258	**0.015** **d = −0.43**	**I**	31.961 ± 6.353	0.085
	**II**	37.285 ± 8.733		**II**	33.576 ± 5.300	
**LVESV** **[mL]**	**I**	51.771 ± 13.705	0.236	**I**	53.761 ± 18.981	0.909
	**II**	50.114 ± 12.858		**II**	53.519 ± 16.384	
**LVEDV** **[mL]**	**I**	105.628 ± 23.173	0.416	**I**	105.569 ± 30.050	0.638
	**II**	108.857 ± 25.132		**II**	113.976 ± 25.660	
**LVEF** **[%]**	**I**	49.857 ± 6.651	**0.023** **r = 0.42**	**I**	53.484 ± 7.082	0.857
	**II**	52.808 ± 8.525		**II**	53.346 ± 7.594	
**Wave E** **[m/s],**	**I**	0.646 ± 0.170	0.132	**I**	0.630 ± 0.157	**0.013** **r = 0.514**
	**II**	0.680 ± 0.182		**II**	0.717 ± 0.193	
**Wave A** **[m/s]**	**I**	0.650 ± 0.160	0.616	**I**	0.645 ± 0.240	0.137
	**II**	0.632 ± 0.207		**II**	0.681 ± 0.228	
**e’ lateral** **[m/s]**	**I**	0.085 ± 0.021	0.063	**I**	0.173 ± 0.182	0.594
	**II**	0.095 ± 0.020		**II**	0.207 ± 0.291	
**e’ septal** **[m/s]**	**I**	0.074 ± 0.022	**0.002** **r = 0.627**	**I**	0.084 ± 0.022	**0.013** **d = 0.52**
	**II**	0.085 ± 0.021		**II**	0.091 ± 0.023	
**E/E’**	**I**	8.375 ± 2.753	0.117	**I**	7.958 ± 4.204	0.602
	**II**	7.641 ± 1.940		**II**	8.316 ± 3.308	
**E/A**	**I**	1.044 ± 0.397	0.134	**I**	1.002 ± 0.419	0.280
	**II**	1.192 ± 0.489		**II**	1.095 ± 0.554	
**TAPSE** **[mm]**	**I**	21.608 ± 4.015	0.587	**I**	24.707 ± 3.586	0.465
	**II**	22.085 ± 5.265		**II**	23.961 ± 3.913	
**MAPSE** **[mm]**	**I**	14.371 ± 2.880	**0.039** **r = 0.428**	**I**	17.076 ± 3.261	**0.043** **r = 0.421**

**LVEDd**—left ventricular end-diastolic diameter [mm], **LVESd**—left ventricular end-systolic diameter [mm], **LVESV**—left ventricular end-systolic volume [mL], **LVEDV**—left ventricular end-diastolic volume [mL], **LVEF**—left ventricular ejection fraction [%], **Wave A**—atrial contraction velocity (diastolic motion during atrial systole) [m/s], **Wave E**—early diastolic ventricular filling velocity [m/s], **e’ lateral**—early diastolic velocity of the lateral mitral annulus [m/s], **e’ septal**—early diastolic velocity of the septal mitral annulus [m/s], **E/E’**—ratio of peak mitral inflow velocity during early diastole to early diastolic mitral annular velocity, **E/A**—ratio of early to atrial mitral inflow velocities, **TAPSE**—tricuspid annular plane systolic excursion [mm], **MAPSE**—mitral annular plane systolic excursion [mm], **d**—Cohen’s d (effect size), **r**—effect size for the Wilcoxon test.

**Table 5:** Intergroup analysis.

	2000 m a.s.l. vs. 3000 m a.s.l.
**LVEDd [mm]**	*p* = 0.604
**LVESd [mm**	*p* = 0.878
**LVESV [mL]**	*p* = 0.584
**LVEDV [mL]**	*p* = 0.901
**LVEF [%]**	*p* = 0.053
**Wave E [m/s]**	*p* = 0.303
**Wave A [m/s]**	*p* = 0.191
**e’ lateral [m/s]**	*p* = 0.161
**e’ septal [m/s]**	*p* = 0.726
**E/E’**	*p* = 0.878
**E/A**	*p* = 0.142
**TAPSE [mm]**	*p* = 0.131
**MAPSE [mm]**	***p* = 0.010, r = 0.33**

**LVEDd**—left ventricular end-diastolic diameter [mm], **LVESd**—left ventricular end-systolic diameter [mm], **LVESV**—left ventricular end-systolic volume [mL], **LVEDV**—left ventricular end-diastolic volume [mL], **LVEF**—left ventricular ejection fraction [%], **Wave A**—atrial contraction velocity (diastolic motion during atrial systole) [m/s], **Wave E**—early diastolic ventricular filling velocity [m/s], **e’ lateral**—early diastolic velocity of the lateral mitral annulus [m/s], **e’ septal**—early diastolic velocity of the septal mitral annulus [m/s], **E/E’**—ratio of peak mitral inflow velocity during early diastole to early diastolic mitral annular velocity, **E/A**—ratio of early to atrial mitral inflow velocities, **TAPSE**—tricuspid annular plane systolic excursion [mm], **MAPSE**—mitral annular plane systolic excursion [mm], **r**—effect size for the Wilcoxon test.

## Data Availability

The data presented in this study are available on request from the corresponding author due to the large volume of results. The data can be made available upon request only.
